# Bioplastic films with unusually good oxygen barrier properties based on potato fruit-juice†

**DOI:** 10.1039/d1ra01178b

**Published:** 2021-03-30

**Authors:** Simi Poulose, Ilari Jönkkäri, Mikael S. Hedenqvist, Jurkka Kuusipalo

**Affiliations:** Faculty of Engineering and Natural Sciences, Materials Science and Environmental Engineering, Tampere University P. O. Box 589 Tampere FI-33014 Finland jurkka.kuusipalo@tuni.fi; Department of Fibre and Polymer Technology, School of Engineering Sciences in Chemistry, Biotechnology and Health, KTH Royal Institute of Technology Stockholm SE-100 44 Sweden mikaelhe@kth.se

## Abstract

In this study, the use of potato fruit juice (PFJ) to make plastic films is presented. PFJ is an interesting raw material as it is obtained as a by-product from the potato-starch industry. The films showed uniquely high oxygen barrier properties, and the PFJ material is therefore a potential replacement for the most commonly used, expensive and petroleum-based ethylene-vinyl alcohol copolymer (EVOH) as a barrier layer in future packaging. The films also exhibit good grease resistance. As expected for hydrophilic materials, they exhibited high water vapour transmission rate, which shows that they, as for EVOH, have to be laminated with hydrophobic polymers in food packaging. The films, having a glass transition temperature between −5 °C and 10 °C, showed elastic–plastic behaviour with stable crack growth.

## Abstract 2

Valorization of potato fruit juice, a potato starch industry by-product, by developing biopolymer films that could replace petroleum based alternatives in packaging, providing a greener and sustainable solution to industrial waste management.

## Graphical abstract

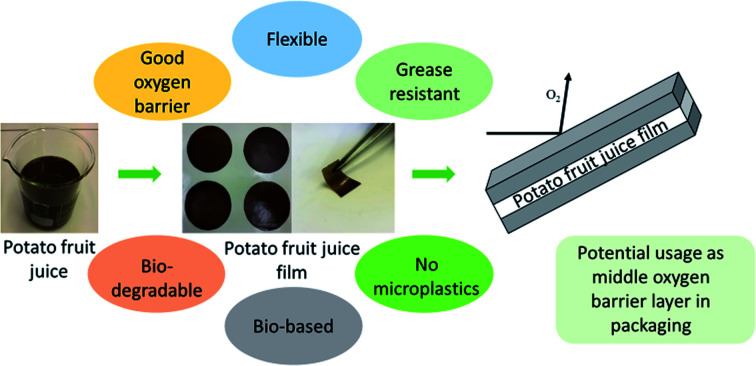

## Introduction

The development of bio-based packaging materials to replace synthetic materials has gained momentum in recent years. This is due to increased awareness about environmental issues caused by green-house gas effects and waste problems associated with petroleum-based materials. Moreover, considerable changes in legislation are forcing industries to shift to natural renewable sources as raw materials for packaging materials. Biopolymers, such as polysaccharides and proteins obtained from biomass, are suitable substitutes to synthetic polymers in several applications, thereby reducing the dependency on petroleum products.^1^

Protein-based materials are suitable in applications, where high moist conditions can be avoided. They are heteropolymers, containing polar and non-polar amino acids, which allow almost unlimited sequential arrangements, facilitating numerous interactions and chemical reactions.^2^ During heat processing, protein denatures and often cross-links *via* specific linkages, *e.g.*, disulfide bonds, which can be exploited for versatile applications.^3^ Moreover, proteins are high barrier materials against permanent gases and hydrophobic compounds under low humidity, and with plasticisers, they can also show good mechanical properties.^4^

A large variety of proteins have been investigated for packaging applications; examples include plant proteins such as wheat gluten,^5,6^ corn zein,^7^ soy protein,^8^ pea proteins,^9^ potato proteins and animal proteins such as casein,^10^ whey,^11^ collagen^12^ and keratin.^13^ The use of a biobased and biodegradable protein has benefits compared to petroleum-based polymers. However, the extend of the benefits depends on how the protein-source is grown and processed and also on the actual petroleum-based polymer to which it is compared to. A clear advantage, even over many of today's commercial biobased and biodegradable plastics, is that the proteins are not directly a problem related to microplastics. They are a nutritional source for soil and animals and also degrade in most environments. Another advantage is that proteins (natural polyamides), are usually processed into plastics at a far lower temperature than polyamides, which lower electric power processing costs, and indirectly, CO_2_ emission from non-renewable electricity. If the protein-source is a by-product or waste from an existing industrial process, then the source is usually cheap, and a more sustainable use of resources is obtained. One such source is potato fruit juice (PFJ), which is obtained as a by-product from the potato-starch industry. It is available in bulk quantities, from 0.7–7 m^3^ per tonne tuber, depending on the technology used in its production.^14^ PFJ is a complex dilute aqueous mixture of numerous different components, and its protein content is approximately 25–30% of the dry matter. In Table S1,† these components are listed, which include proteins, peptides, amino acids and amides, sugars and potassium. It also contains small amounts of other N-containing compounds, organic acids, lipids and phosphorus.^15^

Potato proteins in PFJ have been classified into three groups: patatin (35–40% of total protein), protease inhibitors (25–50%) and other high molecular weight proteins. Patatin consists of a family of glycoproteins with a molecular mass of 40–42 kDa. The protease inhibitors include a wide-range of proteins with molecular masses ranging from 7 to 21 kDa. The other higher molecular weight proteins include starch synthetase, polyphenol oxidase and potato multicystatin inhibitor.^14^

Few studies have been carried out on effectively using potato proteins in food applications.^16,17^ PFJ, being a highly perishable material, for it to be disposed without any environmental concerns, it should be processed properly. Commonly PFJ is used to produce potato protein isolates (PPIs), which find their applications as animal feed and low-quality fertilizers. In addition to that, researchers have investigated the use of PPIs as bio-based polymer films.^18,19^ However, PFJ has not been studied for its film forming properties. By using PFJ, instead of PPI, in bio-based films, the product will be cheaper as it eliminates the step of protein purification/isolation from PFJ. In this study, for the first time, we have shown that it is possible to make plastic films from PFJ and that the properties are promising, even surprisingly good, such as oxygen barrier properties. Films, with and without glycerol plasticisers, are characterized with respect to oxygen-, water- and grease-barrier properties as well as tensile and thermal properties.

## Experimental

### Materials

PFJ was purchased from Finnamyl Oy, Finland, with a dry solids content of 23 wt%, where 31 wt% of the dry content was protein. Glycerol (≥99.5%) was obtained from Sigma-Aldrich Chemie, GmbH, Germany.

### Casting of films

Films were made by casting the PFJ-based material in 14 cm diameter Teflon-coated frying pans bought from IKEA, Tampere, Finland; brand name “Skänka”. Glycerol was added to PFJ in a content equal to 10, 20 and 30 wt% of the protein content of PFJ to yield films with the corresponding names: P10, P20 and P30, respectively. This corresponds to 3, 6 and 9 wt% of the PFJ dry content. The films without glycerol is presented as P0. The content of glycerol was relatively low, but the high content of water/low-molar mass molecules in these made the films too sticky to be handled if higher glycerol contents were used. 20 ml of the PFJ solution was poured into the pans and was swirled to evenly spread in the pan. They were then dried at 40 °C for two days. Then, the films were carefully peeled off from the pans. The thickness of the films was *ca.* 300 μm. All films were conditioned at 40% RH and 23 °C for at least three days before being tested and analysed.

### Thickness measurement

In order to study mechanical and barrier properties, the thickness of the films was measured using a Digimatic Micrometer, Model No. MDE 25 PJ (Mitutoyo 0–25 mm IP65 Coolant Proof Digital Micrometer). The thickness was measured at three random positions and the average was used.

### Water vapour transmission rate (WVTR)

The water vapour transmission rate (WVTR) was obtained *via* a gravimetric method. The developed films were sealed to the open mouth of a test cup of 10 cm diameter using anhydrous calcium chloride as the desiccant. The cups were then stored in a chamber (ESPEC, Model PR-2J, Japan) maintaining 50% RH and 23 °C. These were then weighed two times per day until steady state was reached. Five cups (sample replicates) were used for each film. WVTR was expressed in g per (m^2^ day). The WVTR values were also normalized to thickness to obtain specific water vapour transmission rate (sWVTR) values, which were expressed in g mm per (m^2^ day).

### Oxygen permeability (OP)

The oxygen transmission rate was obtained using a MOCON Analyzer (MOCON OX-TRAN, Model 2/21, MH module) under the testing conditions of 50% RH and 23 °C. The oxygen transmission rate (OTR) was obtained as cm^3^ per (m^2^ day atm). The oxygen permeability was calculated by normalizing the values to film thickness, and is expressed in cm^3^ mm per (m^2^ day atm).

### Tensile testing

Tensile testing was performed to determine tensile modulus, tensile strength and extensibility according to ASTM D882-00 using a Testometric M500 Texture Analyser. Rectangular strips of 15 mm width and 80 mm length were cut from the films. They were then clamped in tensile grips, which were 50 mm apart. The strain rate was 25 mm min^−1^ and a 20 N load cell was used. The tensile modulus E was obtained as the initial slope of stress *versus* the strain curve. Fracture energy was also determined by calculating the area under the stress–strain curve.

### Differential scanning calorimetry (DSC)

A differential scanning calorimeter (Netzsch DSC 214 Polyma) was used to determine the glass transition temperature (*T*_g_) of the developed films. Samples weighing 5–10 mg were placed in aluminium pans with pierced lids, which were then sealed. The empty aluminium pan with a pierced lid was used as the reference. The samples were heated from −50 to 200 °C at the rate of 20 °C min^−1^. The glass transition temperature (*T*_g_) was determined as the midpoint of the baseline shift. All measurements were made in triplicates.

### Thermogravimetric analysis (TG)

Thermogravimetric analysis was carried out using a Netzsch TG 209 F3 Tarsus thermogravimetric analyser. Samples of approximately 10 mg were heated from 30 °C to 500 °C at a heating rate of 5 °C min^−1^. Three replicates were used.

### Scanning electron microscope (SEM)

A JEOL JSM-IT500 variable pressure scanning electron microscope was used to investigate the surface morphology of the film. The samples were coated with a thin gold layer. The high vacuum study was carried out at 5 kV acceleration voltage using a secondary electron detector.

### Grease resistance

Grease resistance was measured according to ASTM-F119-82. Fluorescent thin layer chromatography (TLC) sheets were used to detect the penetrated grease. Films were placed on the TLC sheet marked with test points. Then, two flannel pieces were placed on each test point with weights on them, and the whole assembly was placed in an oven at 40 °C for 30 min. It was subsequently taken out and after removing the weights, six drops of olive oil were pipetted on the flannel piece. Then, the weights were re-assembled and the setup was put back in the oven at 40 °C. To examine the grease penetration onto the TLC sheets, the sample assembly was taken out carefully from the oven at set intervals. The weights and sample film were removed carefully, and the TLC sheets were examined under UV light for any possible penetration marks, which were detected with darkening or colour change on the TLC sheets. After examination, the sample assembly was placed in the oven again for further sampling. The time taken for oil penetration for each test points was noted. Five test points were used for each film.

### Fourier transform infrared spectroscopy (FTIR)

The spectra were collected between 600 and 4000 cm^−1^ using an FTIR-ATR Spectrum One (Perkin Elmer). The number of scans used was 64 and the resolution was 4 cm^−1^.

## Results and discussion

### Overall film characteristics

**Fig. 1:**
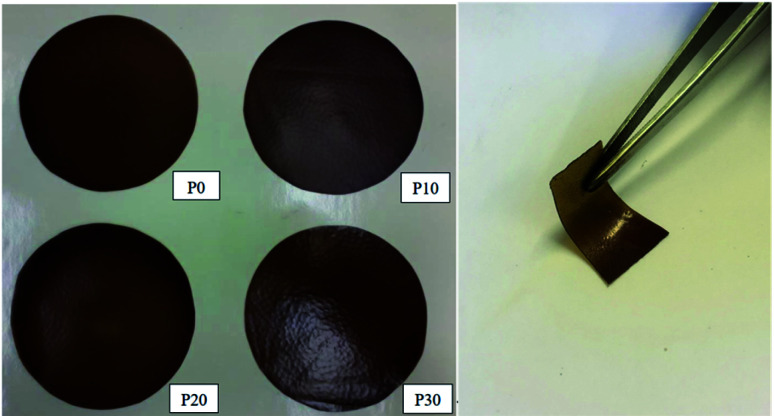
Developed PFJ films (left) and a bent P20 film (right).

All films were easy to peel off from the mould and they showed good cohesion and no brittleness. The films obtained with all compositions are shown in Fig. 1. Glycerol is a proven efficient plasticizer for proteins^3^ and is also compatible with PFJ. It was observed that even the film without glycerol was flexible due to the presence of water and other small molecules with plasticising abilities (Table S1†).

**Fig. 2:**
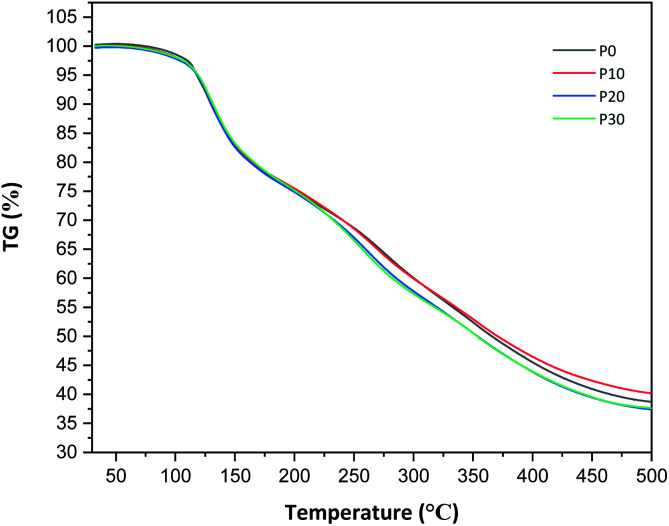
TG curves of the protein films conditioned at 40% RH and 23 °C.

**Fig. 3:**
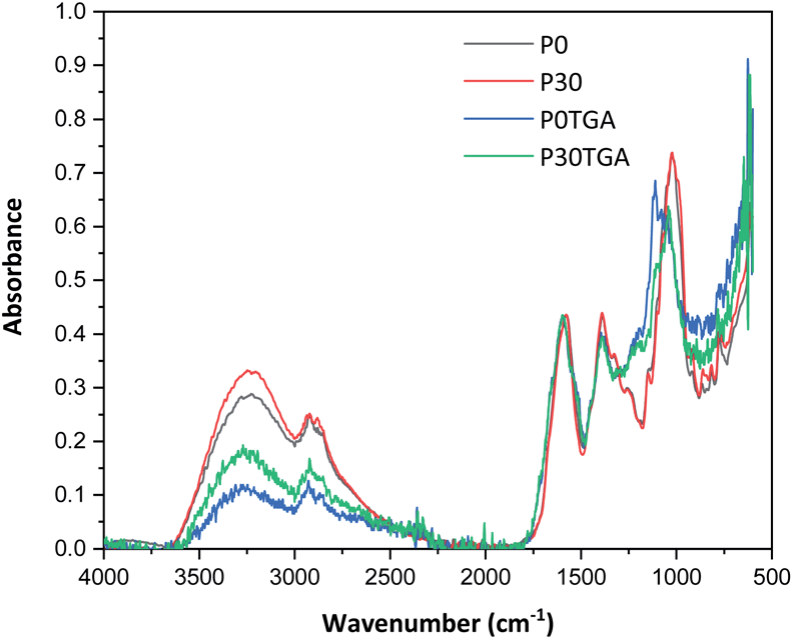
FTIR spectra of films conditioned at 40% RH and 23 °C (P0, P30) and directly after being heated from 30 to 150 °C at a rate of 5 °C min^−1^ (P0TGA, P30TGA). The IR absorbance was normalized to the peak starting from *ca.* 1570 cm^−1^ to *ca.* 1600 cm^−1^, which includes the amide I peak.

**Fig. 4:**
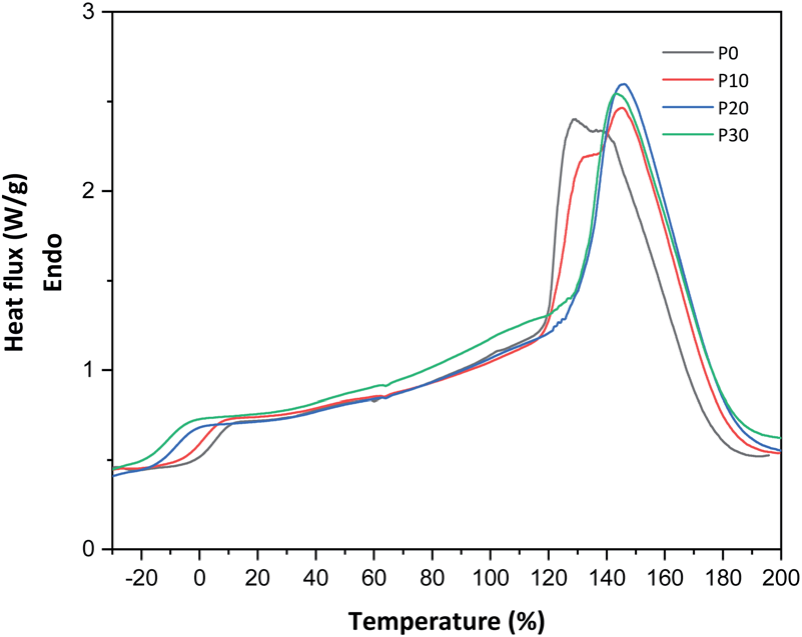
DSC curves of developed PFJ films.

As observed in the TG curves in Fig. 2, the films contained *ca.* 2.5 wt% of loosely attached water, as indicated by the weight loss up to slightly above 110 °C. A large loss in weight was observed between this temperature and up to *ca.* 150 °C. This was due to the evaporation of more strongly bonded water and possibly other volatile species from the complex mixture of components in PFJ (Table S1†). The total weight loss up to 150 °C was 15.7 ± 0.5 (P0), 15.1 ± 0.4 (P10), 14.5 ± 0.2 (P20), 14.4 ± 0.1 (P30) wt%. As observed in the FTIR spectra (Fig. 3), the broad absorption centred at *ca.* 3250 cm^−1^ decreased significantly when the films were heated to 150 °C. The absorbance was due to the presence of water, glycerol and other O–H and/or N–H (absorbance due to these bond vibrations) containing compounds. The DSC curves confirmed a two-step loss of volatiles before 200 °C (a gradual increase in the endothermal heat flux from room temperature, ascribed mainly to water, which developed into a large endothermal heat flux, peaking somewhere between 120 and 160 °C (Fig. 4)). The small difference between the TG curves at 230 °C, and higher, was due primarily to the evaporation of glycerol (Fig. 2). It should be noted that there is a large char residue of the samples after the TG experiment, which is often observed for proteins,^20^ and indicates that the fire resistance is better than pure hydrocarbon polymers. The presence of water in the material is also adding to this property.

The glass transition temperature, obtained from DSC, for samples P0, P10, P20 and P30 were 10.7 °C ± 1.5, 2.6 °C ± 0.1, −3.7 °C ± 5.6 and −5.4 °C ± 0.2, respectively (Fig. 4). Due to the presence of water and other low-molar mass polar “plasticising” components, even the film without glycerol had a relatively low glass transition temperature. This explains the film flexibility. The *T*_g_ decreased further, as expected, with the increase in the glycerol content. A similar decrease in the glass transition temperature is reported by Galietta *et al.*^21^ on whey protein-based films using glycerol used as the plasticizer.

**Fig. 5:**
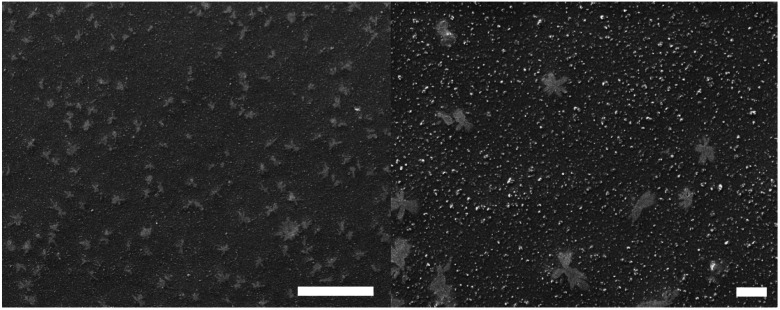
The surface morphology of the PJF P0 film. The scale bar represents 100 μm in the left figure and 10 μm in the right figure.

The SEM images of the PFJ film surface are shown in Fig. 5. The film had a pore-free surface with dendritic structures distributed randomly over its surface. The dendritic structures were present in all films, and the patterns indicated the presence of one or several components that crystalized during the film formation. There are several components in the heterogeneous PFJ that can potentially crystallize, including lipids and citric acid.

### Oxygen permeability

The oxygen permeability (OP) of the PFJ films are shown in Table 1. The values were unexpectedly low, considering the testing conditions (50% RH). Overall, the values also decreased with the increase in the glycerol content. This may be related to an increase in the uniformity of the films with the addition of glycerol. At the overall low content of the added plasticiser, here it is also likely that the increase in hydrogen bonding dominates over plasticisation and then reduces OP. It is well-known that hydrogen bonding leads to reduced gas barrier properties at low or intermediate relative humidity.^22^

**Table 1:** Oxygen barrier properties

Sample	OTR cm^3^ per (m^2^ day atm)	OP cm^3^ mm per (m^2^ day atm)
P0	0.042 ± 0.022	0.014 ± 0.010
P10	0.029 ± 0.024	0.009 ± 0.006
P20	0.033 ± 0.006	0.010 ± 0.001
P30	0.025 ± 0.004	0.008 ± 0.001

This unusually good oxygen barrier property may be due to the crystals formed on the films, as shown in Fig. 5. It is expected that these crystals are impermeable and hence contribute to high barrier properties.

However, the result is in contrast to those obtained in the studies conducted on potato-protein-isolate (PPI) films by Schäfer *et al.*,^18^ where the OP values showed a linear increase with the addition of glycerol. The plasticiser content was significantly higher (100 to 140)/100 w/w glycerol/protein (PPI: 91.3% protein dry basis)compared to that used in our study. Notably, their OP values were also significantly higher than those obtained here (23–39 cm^3^ mm per (m^2^ day atm)). Furthermore, in a study conducted in peanut protein films using glycerol as the plasticizer, Jangchud and Chinnan^23^ reported that there was no effect of glycerol concentration (0.67–1.67 g g^−1^ of protein) on the OP values of the films.

The OP value of the PFJ films was indeed comparable to that of high-barrier polymers. The OP values of ethylene-vinyl alcohol (EVOH) with 32 and 44 mol% ethylene are 0.01 and 0.04 cm^3^ mm per (m^2^ day atm), respectively, at 20 °C and 65% RH.^24^ For further comparison, the OP values of polyethylene terephthalate (PET) and low-density polyethylene (LDPE) obtained are 1.14 and 68.5 cm^3^ mm per (m^2^ day atm) under the same conditions.^24^

### Water vapour transmission rate

The WVTR and sWVTR of the PFJ films with different contents of the plasticizer are presented in Table 2. In contrast to the OP data, the water vapour transmission rate increased with an increase in the plasticizer content. Since OP and sWVTR were not following the same trend, the main reason for the increase in sWVTR with an increase in the glycerol content was most likely due to the increase in hydrophilicity/polarity with increasing glycerol content.^25^

**Table 2:** Water vapour barrier properties of PFJ films

Sample	WVTR g per (m^2^ day)	sWVTR g mm per (m^2^ day)
P0	15.7 ± 2	4.8 ± 0.5
P10	21.0 ± 2	6.9 ± 0.5
P20	27.8 ± 3	9.2 ± 0.6
P30	37.8 ± 6	13.3 ± 2

A similar reducing trend in water-vapour barrier properties with increasing amount of the plasticizer is reported for gelatin films by Sobral *et al.*^26^ and for wheat gluten films by Gontard *et al.*^27^ Kowalczyk and Baraniak^25^ also stated that an increase in the glycerol content in pea protein isolate films had caused an increase in the WVP of the films. Schäfer *et al.*^18^ obtained sWVTR values between 79 and 134 g mm per (m^2^ day) for PPI-based films, which are, again, as in the case of OP, significantly higher than those obtained in this study. This is explained by their significantly higher plasticizer content.

Nevertheless, and as expected for a protein material, the water vapour permeability of the present films was higher than that of well-known water barrier materials such as LDPE (0.45 g mm per (m^2^ day) at 90% RH and 40 °C) and polyimide (0.06–1.35 g mm per (m^2^ day) 50% RH and 23 °C).^24^

### Grease resistance

The TLC sheets were not stained with oil, even after 2 weeks of exposure. This indicated that the samples had good grease resistance (as observed with the current thickness). In a similar study, Park *et al.*^28^ investigated the grease resistance of a soy protein isolate (SPI)-coated paper *via* a modified TAPPI test T-507. They reported that the grease resistance of these papers with an SPI coating thickness on paper greater than 12.1 μm was comparable to polyethylene laminated papers, which are used in sandwich packaging in fast food restaurants.

### Mechanical properties

**Fig. 6:**
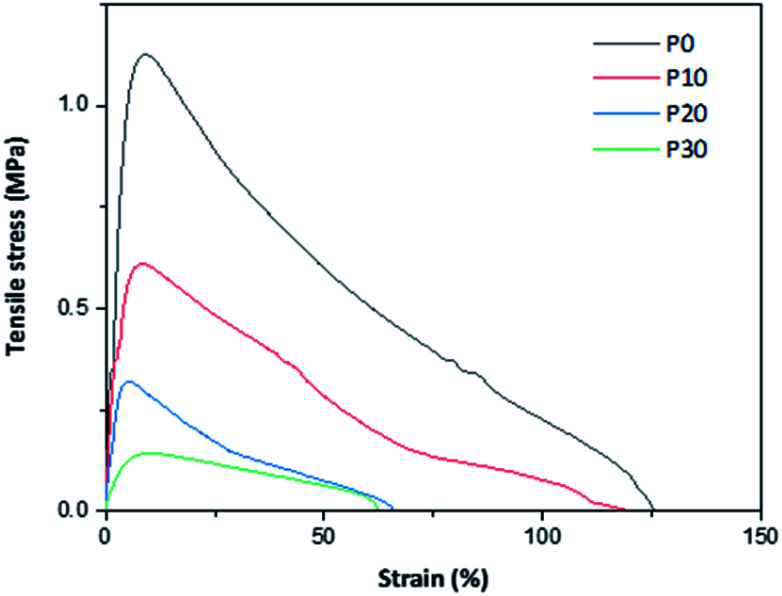
Examples of tensile stress–strain curves of the PFJ films.

Representative tensile stress–strain curves are given in Fig. 6 and calculated parameters are given in Table 3. The curve-shapes were typical for materials following elastic–plastic fracture mechanics (EPFM) with a stable crack growth.^29^ All the mechanical parameters decreased with the increase in the plasticizer content. The decrease in strength and stiffness was expected, but the decrease in extensibility is somewhat unexpected. However, the extensibility is quite high already for the glycerol-free material and the decrease in extensibility can be seen as a weakening of the material (as observed by the lower strength). In fact, a similar trend in mechanical properties is reported by Schäfer *et al.*,^18^ who observed a decrease in strength from 2 to 0.6 MPa, a decrease in modulus from 48 to 14 MPa and a decrease in extensibility from ∼12 to 6% for PPI films, when the plasticiser content is increased from 100 to 140% (w/w protein). Similarly, Shaw *et al.*^30^ reported that when xylitol is used as a plasticizer in whey protein isolate (WPI) films, the tensile strength, modulus and extensibility decreases with an increase in the plasticizer content. However, a different trend is observed in these films when glycerol and sorbitol are used as plasticizers. The tensile strength and tensile modulus are reported to decrease, whereas the extensibility increases with the increase in the amount of added plasticizer.

**Table 3:** Mechanical properties of PFJ films

Sample	Strength (MPa)	Modulus (MPa)	Fracture strain (%)	Fracture energy (MJ m^−3^)
P0	1.20 ± 0.11	18.7 ± 5.1	105 ± 27	59.1 ± 6.0
P10	0.54 ± 0.06	8.1 ± 0.6	95 ± 20	30.0 ± 2.2
P20	0.31 ± 0.02	5.5 ± 0.5	75 ± 8	10.8 ± 1.4
P30	0.16 ± 0.02	2.4 ± 0.5	72 ± 15	6.3 ± 0.3

## Conclusions

This is the first study to show that potato fruit juice can be used to make flexible cohesive films. The films were flexible even without any added plasticiser, and showed unexpectedly good oxygen barrier properties. Due to the hydrophilicity, the films had also a good grease resistance. The water vapour permeability was found to increase with added plasticizer, whereas the oxygen permeability decreased. The former effect was explained by the increased polarity of the films, whereas the latter is due, most likely, to an increasing uniformity of the films with the addition of a plasticizer. Moreover, the tensile properties and glass transition temperature also showed a decreasing trend with the increase in the plasticizer content. The results are unique and show the potential of PFJ as a source for future plastic films such as in packaging. It has the potential as an oxygen barrier layer in food packaging and as a replacement for the expensive and petroleum-based EVOH, which is the most common barrier-layer material in packaging. Both PFJ and glycerol are by-products (the latter from biodiesel production) and the implementation of these in packaging will lead to more sustainable plastics and a more sustainable use of resources. It will also reduce the problem associated with microplastics.

## Conflicts of interest

There are no conflicts of interest to declare.

## Supplementary Material

RA-011-D1RA01178B-s001

## References

[cit1] Zhong Y., Godwin P., Jin Y., Xiao H. (2019). Adv. Ind. Eng. Polym. Res..

[cit2] Cuq B., Gontard N., Guilbert S. (1998). Cereal Chem..

[cit3] Hernandez-Izquierdo V. M., Krochta J. M. (2008). J. Food Sci..

[cit4] Gómez-Estaca J., Gavara R., Catalá R., Hernández-Muñoz P. (2016). Packag. Technol. Sci..

[cit5] Lens J. P., De Graaf L. A., Stevels W. M., Dietz C. H. J. T., Verhelst K. C. S., Vereijken J. M., Kolster P. (2003). Ind. Crops Prod..

[cit6] Roy S., Gennadios A., Weller C. L., Testin R. F. (2000). Ind. Crops Prod..

[cit7] Li Y., Bai Y., Huang J., Yuan C., Ding T., Liu D., Hu Y. (2020). J. Food Eng..

[cit8] dos Santos Paglione I., Galindo M. V., de Medeiros J. A. S., Yamashita F., Alvim I. D., Ferreira Grosso C. R., Sakanaka L. S., Shirai M. A. (2019). Food Packag. Shelf Life.

[cit9] Kowalczyk D., Gustaw W., Zieba E., Lisiecki S., Stadnik J., Baraniak B. (2016). Food Hydrocoll..

[cit10] Picchio M. L., Linck Y. G., Monti G. A., Gugliotta L. M., Minari R. J., Alvarez Igarzabal C. I. (2018). Food Hydrocoll..

[cit11] Cruz-Diaz K., Cobos Á., Fernández-Valle M. E., Díaz O., Cambero M. I. (2019). Food Packag. Shelf Life.

[cit12] Xu J., Liu F., Goff H. D., Zhong F. (2019). Food Hydrocoll..

[cit13] Alashwal B. Y., Saad Bala M., Gupta A., Sharma S., Mishra P. (2020). J. King Saud Univ., Sci..

[cit14] Løkra S., Strætkvern K. (2009). Food.

[cit15] van Koningsveld G. A. , Physico-chemical and functional properties of potato proteins, 2001

[cit16] Knorr D. (1977). J. Food Sci..

[cit17] Schmidt J. M., Damgaard H., Greve-Poulsen M., Larsen L. B., Hammershøj M. (2018). Food Hydrocoll..

[cit18] Schäfer D., Reinelt M., Stäbler A., Schmid M. (2018). Coatings.

[cit19] Newson W. R., Rasheed F., Kuktaite R., Hedenqvist M. S., Gällstedt M., Plivelic T. S., Johansson E. (2015). RSC Adv..

[cit20] Blomfeldt T. O. J., Nilsson F., Holgate T., Xu J., Johansson E., Hedenqvist M. S. (2012). ACS Appl. Mater. Interfaces.

[cit21] Galietta G., Di Gioia L., Guilbert S., Cuq B. (1998). J. Dairy Sci..

[cit22] Hedenqvist M. S. , in Environmental Degradation of Materials, ed. M. Kutz, Elsevier/William Andrew Publ., Oxford, UK, 3rd edn, 2018, pp. 559–581

[cit23] Jangchud A., Chinnan M. S. (1999). LWT--Food Sci. Technol..

[cit24] Massey L. K. , Permeability Properties of Plastics and Elastomers - A Guide to Packaging and Barrier Materials - Plastics Design Library, 2nd edn, 2003

[cit25] Kowalczyk D., Baraniak B. (2011). J. Food Eng..

[cit26] Sobral P. J. a., Menegalli F. C., Hubinger M. D., Roques M. a. (2001). Food Hydrocoll..

[cit27] Gontard N., Guilbert S., Cuq J. (1993). J. Food Sci..

[cit28] Park H. J., Kim S. H., Lim S. T., Shin D. H., Choi S. Y., Hwang K. T. (2000). J. Am. Oil Chem. Soc..

[cit29] Deformation and Fracture Behaviour of Polymers, ed. W. Grellmann and S. Seidler, Springer Berlin Heidelberg, Berlin, Heidelberg, 2001

[cit30] Shaw N. B., Monahan F. J., O'Riordan E. D., O'Sullivan M. (2002). J. Food Sci..

