# Supplementary material for: Bioplastic films with unusually good oxygen barrier properties based on potato fruit-juice
Source: RSC Adv. 2021 Mar 30;11(21):12543–8. doi: 10.1039/d1ra01178b (PMC8697038; doi:10.1039/d1ra01178b)
Supplement: RA-011-D1RA01178B-s001 [file RA-011-D1RA01178B-s001.pdf]

Electronic Supplementary Material (ESI) for RSC Advances.
This journal is © The Royal Society of Chemistry 2021

Electronic Supplementary Information

Bioplastic films with unusually good oxygen barrier properties, based on potato fruit-juice

Simi Poulose[a], Ilari Jönkkäri[a], Mikael S. Hedenqvist[b,*], Jurkka Kuusipalo[a,*]

[a] Faculty of Engineering and Natural Sciences, Materials science and environmental engineering, Tampere University, P. O. Box 589, FI-33014, Tampere, Finland.

[b] Department of Fibre and Polymer Technology, School of Engineering Sciences in Chemistry, Biotechnology and Health, KTH Royal Institute of Technology, SE-100 44 Stockholm, Sweden.

Table S1 Average composition of potato fruit juice (dry matter) [1]

Component	% drymatter
Protein (N x 6.25)	26.8
Peptides (N x 6.25)	4.4
Amino acids + amides (N x 5.13)	9.6
Other N-containing compounds	1.8
Sugars	15.8
Lipids	2.2
Citric acid	10.0
Ascorbic acid	0.6
Other organic acids (e.g. malic and pyrolidone carboxylic acid)	2.6
Chlorogenic acid	0.4
Caffeic acid	0.1
Potassium	11.2
Phosphorus	1.0
Other components	10.1

REFERENCE

1 G. A. van Koningsveld, *Physico-chemical and functional properties of potato proteins*, 2001.
